# A Cinnabar Case

**Published:** 1889-09

**Authors:** E. W. Berridge

**Affiliations:** London


### A CINNABAR CASE.

E. W. Berridge, M. D., London.

May 14th, 1889.—Mrs. —— complained of the following symptoms when walking: a subjective sensation as if something protruded in left groin, and the left leg feels shorter than the right one. Both great toes feel strained, as if they would go out of joint. Left knee, hip, and calf stiff, relieved by urinating. These symptoms she had had for about a week, but the pain in groin she had suffered from at intervals for some years. *Cinnabar*$^{cm}$ (Fincke), one dose.

May 17th.—Reports that all the symptoms went next day, but returned in evening. Last evening they were as bad as ever. *Cinnabar*$^{cm}$ (Fincke), one dose.

May 21st.—The symptoms were at first aggravated, especially on 19th. Yesterday they improved after mid-day. To-day still further improvement, but the full feeling in left groin is unchanged.

May 28th.—Reports that on 22d was quite well; went to Exeter Hall and had to stand for three hours, but without return of the symptoms. The fullness in groin lasted a little longer than the other symptoms.

June 7th.—Reports that there has been no return.

*Cinnabar* has feeling of shortening of *left* leg in *walking*.

*Causticum* has feeling of shortening of *right* leg on *rising*.

*Carbo animalis* has feeling of elongation of *right* leg at night on lying down.

*Thuja* has feeling of elongation of *left* leg.

Allen's *Index* omits the *Thuja* symptom, and the right-sided action of *Causticum* (see pp. 717, 719).

In the same patient, *Agaricus muscarius*$^{99m}$ (Fincke) removed an objective and subjective coldness of nates.

*Lachesis*$^{3)}$ removed a stinging as if a hot needle were thrust into tip of tongue.

The case of iritis cured by *Lac felinum*, reported at pp. 192–4 Vol. IX of Homœopathic Physician still remains well, June, 1889.

